# A New Method for the Follow-Up of Patients with Alopecia Areata

**DOI:** 10.3390/jcm13133901

**Published:** 2024-07-03

**Authors:** Giulio Bortone, Gemma Caro, Lorenzo Ala, Luca Gargano, Alfredo Rossi

**Affiliations:** 1 Dermatology Unit, Department of Clinical Internal, Anesthesiological and Cardiovascular Science, Sapienza University of Rome, 00185 Rome, Italyalfredo.rossi@uniroma1.it (A.R.); 2 Dermatology-Ente Ospedaliero Cantonale (EOC), Ospedale Regionale di Bellinzona e Valli, 6500 Bellinzona, Switzerland

**Keywords:** alopecia areata, telemedicine, teledermatology, trichology

## Abstract

**Background:** Teledermatology is the application of information and telecommunication technologies in the field of dermatology to provide remote care services based on the exchange of clinical information within a network of professionals or between professionals and patients. Tele-trichoscopy is a non-invasive, inexpensive, and easy-to-use method that applies the principle of surface microscopy at different magnifications. Alopecia areata is a non-scarring alopecia with a chronic evolution, which often needs a close follow-up. **Objectives:** The aim of our work was to analyze the possible benefits of telemedicine for the follow-up of patients with alopecia areata. **Materials and Methods:** We enrolled patients with alopecia areata, identified during the first trichological evaluation, and we divided the patients into two groups. One group was provided with the devices necessary for a telemedicine follow-up. The other group had a conventional follow-up. The total follow-up time was one year. All of the patients’ photos were blindly evaluated by a team of expert dermatologists who were asked to indicate the quality of the images. The photos were also evaluated to identify disease markers. During the follow-up period, questionnaires were administered to both groups of patients to assess the quality of the visit, the level of patient satisfaction, and to identify any issues encountered by the patients. **Results:** There was a high degree of concordance between the images obtained by outpatient trichoscopy and those obtained by telemedicine. The satisfaction levels were the same between the patients followed via telemedicine and those seen in the outpatient clinic. **Conclusions:** Telemedicine applied to trichology using trichoscopy was proven to be a valid system for managing the follow-up of patients suffering from chronic recurrent scalp diseases and, above all, for maintaining continuity of care.

## 1. Background

Alopecia areata is one of the most common forms of non-scarring alopecia. It has an immunological, polygenic, and multifactorial pathogenesis and a chronic recurrent course. In 10–42% of cases, there is a familial predisposition, leading to a 2% higher risk of developing the condition over a lifetime compared to the general population [1]. While this condition does not affect the patient’s life directly, it can have serious repercussions on their psychological well-being, affecting their social life. The etiopathogenetic mechanisms responsible for alopecia areata are still largely unknown. Genetic, infectious, hormonal, vascular, and psycho-neuro-immuno-endocrine factors have been hypothesized, although current literature data attribute a significant role to immune system alterations. This hypothesis is supported by the frequent association with other autoimmune diseases such as vitiligo, Hashimoto’s thyroiditis, and celiac disease; by the overexpression of class I and II HLA in the lower part of the hair follicle; by the presence of lymphocytic alterations at the serum level; and by the frequent detection of autoantibodies. In alopecia areata, the main targets are the follicles in the early stages of anagen because these structures are more vulnerable at this stage [2,3].

In the diagnosis of alopecia areata, trichoscopy is essential in order to detect the disease markers. These patterns are represented by black dots, yellow dots, exclamation mark hairs (tapering hairs), and gray dots. These findings should always be sought at the center and edge of the alopecic patch, and they are crucial to determining the phase of the disease. The presence of black dots signifies active disease, often associated with tapered hairs and broken hairs. Exclamation mark hairs indicate a subacute phase, meaning that the process has occurred and is progressing in its natural evolution. The frequency of yellow dots increases in chronic forms of AA, where they are very numerous and have a regular distribution; this trichoscopic sign is highly indicative of a diagnosis of AA, but is not very specific because it may be present in other types of alopecia. In the regrowth phase, upright, regrowing, depigmented, and tapered hairs can be observed, which transform into white terminal hairs that will eventually undergo pigmentation. Rarely, growth occurs with terminal and pigmented hairs; it is not uncommon to find the presence of circle hairs, pig tail hairs, Pohl–Pinkus, and zigzag hairs. These findings could also be present in hair outside of the scalp, for example, in the eyebrows and beard [4,5,6,7].

Recently, the advent of telemedicine has revolutionized the approach to patient management. Telemedicine enables remote consultation between the physician and patients, allowing for accurate assessment and regular monitoring without the need for physical appointments. This innovative approach not only improves access to medical services, but may also reduce waiting times for diagnosis and treatment, enhancing the overall clinical outcome for patients [8,9].

### Objective

This study arises from the need to implement telemedicine in trichoscopy, given the limited evidence currently available. It seeks to demonstrate the comparable validity between images captured during physical examinations and those sent remotely.

## 2. Materials and Methods

We enrolled patients aged between 18 and 60 years old who were suffering from alopecia areata during their first visit to our trichology clinic. We divided the patients into two groups: one group received a follow-up through conventional outpatient care, while the other was followed up via telemedicine.

The total follow-up period was set at 1 year. During the first visit in the outpatient clinic, consent for the publication and transmission of images was obtained, along with acceptance of the privacy policy and terms of service.

To enable telemedicine visits and standardized, reproducible follow-ups, a chatbot capable of dispatching automated messages tailored to individual patient requirements was created (the major details are indicated in Table 1).

**Figure 1:**
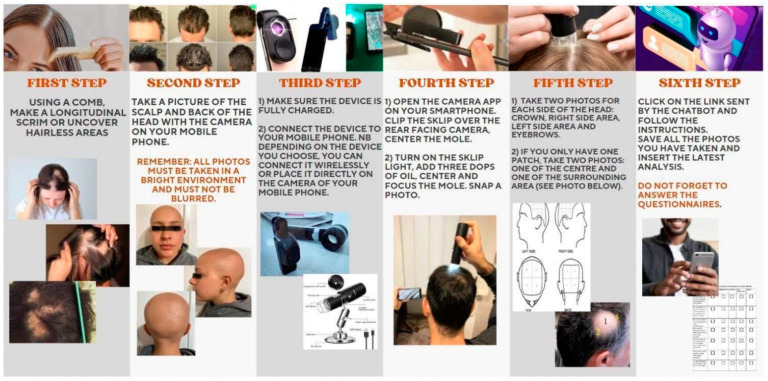
Procedure instructions provided to patients during the initial visit to ensure correct follow-up.

The patients assigned to the telemedicine follow-up were adequately briefed regarding the nature of the visit and the objectives of the service. A medical record was created, after which a patient’s univocal code was generated. Compliance with patient privacy regulations was of utmost importance, requiring the assignment of a unique code to each patient during initial consultations to ensure secure data management. This coding system allowed the bot to link patient communications with their respective codes, thereby protecting patient confidentiality. The entire bot-messaging procedure is reported in Table 2. The patient was given a micro-camera and instructed on its correct use; this was made possible through our procedure instructions, which were illustrated in the form of flowcharts and provided to each patient (Figure 1).

During the first visit, the patient sends the univocal code to the chatbot’s contact. The bot then sends a welcome message to the patient and summarizes the different steps needed to ensure a proper follow-up. These messages perform various functions, including inquiries about completed diagnostic tests, completion of quality-of-life assessments, and the solicitation of monthly photographs to evaluate treatment progress. By keeping the patients informed about their treatment and ensuring their adherence to medical recommendations, our bot serves as a valuable tool in patient management.

Moreover, the bot is equipped to address common patient queries, offering insights into medical conditions, treatment alternatives, and contraindications. This feature fosters patient engagement and enhances their comprehension of their condition, empowering them to actively participate in their care.

Two devices were used for tele-trichoscopy: A Lightswim wireless/USB digital microscope (Apex Ce Specialists Limited, Cork, Ireland) with 50×–100× magnification, and a Sklip^®^ microlens microscope with 6 LCD lights and with 10×–50× magnification. The digital microscope uses two connection systems: wireless, or via USB-C cable or Lightning Cable. Sklip^®^ (Lake Oswego, OR, USA) is a type of dermatoscope that is positioned directly in front of the cell phone camera; when correctly aligned with the phone’s camera, the device allows images of the skin’s surface to be taken with greater definition and resolution, with the possibility of analyzing the image using dedicated software, thus proving to be a valid imaging method.

The photos of the patients followed in the clinic were taken with a video dermatoscope (FotoFinder^®^, Bad Birnbach, Germany).

All of the photos were blindly evaluated by a team of expert dermatologists. They were asked to indicate the quality of all of the images (both those received via telemedicine and those acquired in the clinic) and their usability for diagnosis and follow-up.

The photos were also evaluated to identify disease markers in order to determine the disease phase and to decide on the continuation of treatment.

In the case of low-quality images, users were invited to submit new ones to obtain a reliable assessment.

During the follow-up period, questionnaires were administered to both groups of patients to assess the quality of the visit and the level of patient satisfaction, and to identify any issues encountered.

## 3. Results

A total of 60 patients with alopecia areata of the scalp were enrolled. Of these, 39 were women and 21 were men aged between 18 and 60 years, with an average age of 28 years.

Of the 30 patients who were followed via telemedicine, all participants completed the study. Of these, 90% completed the follow-up period via telemedicine, while 10% required an outpatient visit due to difficulties in image acquisition; 70% completed the entire planned follow-up period without the need to change their therapy. Additionally, 20% of patients, despite needing to change their therapy, still completed the entire follow-up period remotely.

Of the 30 patients who were followed in the clinic, 80% completed the follow-up period adequately. Of these patients, 16.7% did not perform all of the follow-ups stipulated in the follow-up plan, and 3.33% attended only the first visit.

The analysis of the questionnaires on the quality of the visit reveal similar satisfaction levels between patients who were followed via telemedicine and those who were evaluated in the outpatient clinic. In particular, 93.33% of outpatients and 90% of telemedicine patients were very satisfied. A significant proportion of patients followed via telemedicine (96.66%) reported significant time savings, while 93.33% were highly satisfied with the speed of communication with their healthcare providers.

**Figure 2:**
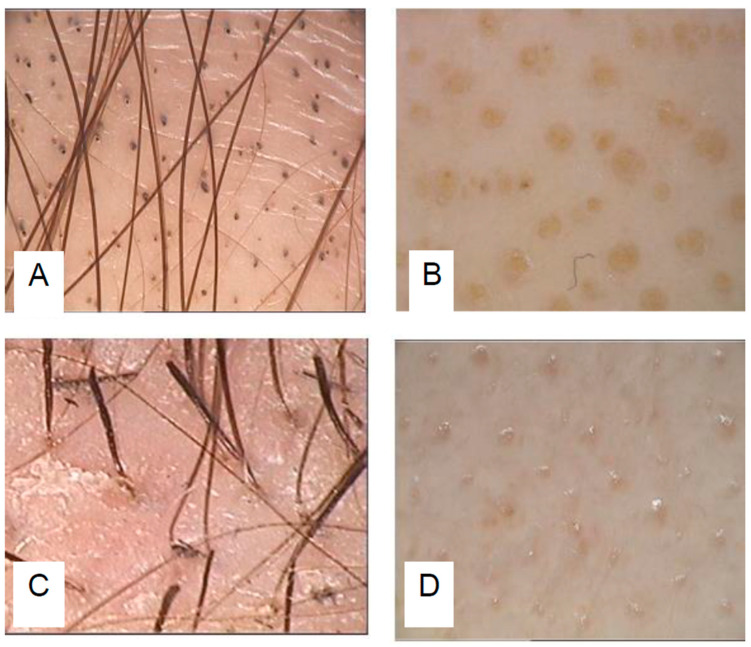
Images taken with traditional trichoscopy showing black dots (**A**), yellow dots (**B**), exclamation mark hairs (**C**), and gray dots (**D**).

**Figure 3:**
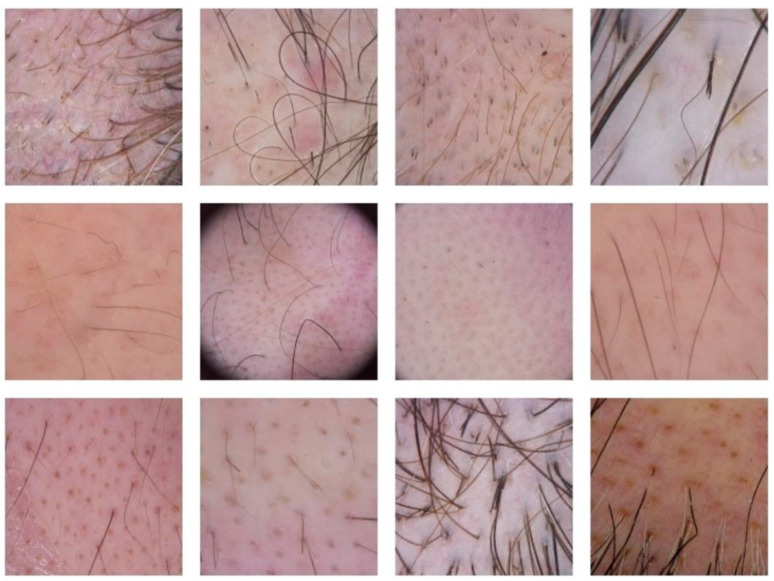
Images received from patients followed via telemedicine.

Over the course of this study, we received a substantial number of trichoscopic photographs (Figure 2 and Figure 3). Most of these photographs were deemed to be of sufficient quality to enable accurate diagnosis. The clarity and detail captured in these images allowed our experts to identify various clinical signs associated with alopecia areata, such as exclamation mark hairs, black dots, and yellow dots. The Sklip^®^ Dermatoscope has been deemed superior by our team in capturing the diverse array of signs associated with alopecia areata, as compared to its counterpart, the Lightswim Dermatoscope.

## 4. Discussion

In the field of telemedicine, patient follow-up is an area of growing interest and importance. Numerous studies have investigated the effectiveness and acceptability of remote follow-up in a variety of clinical settings. However, despite significant advances in the field, there are still challenges and unanswered questions regarding the optimization of follow-up services in telemedicine [10,11]. Our project endeavors to apply telemedicine to trichology, necessitating a user-friendly approach in order to accommodate the active role expected of patients during remote visits and to fulfill the aspiration of expanding this approach extensively. On the basis of the data presented, our study demonstrates that telemedicine applied to trichology in the context of chronic recurrent diseases, such as alopecia areata, is a technique with great potential for development. The data are extremely encouraging and provide us with important insights into how new technologies can enhance patient care.

It is important to highlight the high completion rate of patient follow-ups. Among the 30 patients followed via telemedicine, 27 completed the entire follow-up period. This result is notable, particularly considering that only three patients required an outpatient visit due to difficulties in image acquisition. In comparison, 24 patients followed in the clinic adequately completed their follow-ups. However, it is important to note that five of these patients did not perform all of the stipulated follow-ups, and one patient attended only the first visit. These data demonstrate a strong adherence to the telemedicine program, highlighting its effectiveness in keeping patients engaged in their care journey. Turning to patient satisfaction, the analysis of the questionnaires reveals nearly identical satisfaction levels between those who were followed via telemedicine and those evaluated in the clinic. In total, 93.33% of outpatient clinic patients reported being very satisfied, a figure comparable to the 90% of patients followed via telemedicine. This indicates that telemedicine can provide a quality of care equivalent to that offered during in-person visits. A particularly interesting aspect of our study is the time savings reported by patients. A significant 96.66% of patients followed via telemedicine reported considerable time savings, while 93.33% expressed high satisfaction with the speed of communication with their healthcare providers. These data demonstrate that telemedicine not only improves access to care, but also optimizes the interactions between patients and doctors, making the process more efficient and convenient for both parties. Another crucial aspect of our study was the identification of devices that can serve as viable alternatives to traditional in-clinic trichoscopy, and how, with adequate training, patients can actively contribute to their diagnostic and therapeutic journey. Alopecia, particularly alopecia areata, is a condition that significantly impacts patients’ quality of life. Effective management of this pathology requires continuous and detailed monitoring of the scalp and hair condition. Traditionally, this monitoring occurs in a clinical setting through in-clinic trichoscopy. However, this approach can be demanding for patients in terms of time and accessibility of care. The benefits of the telemedicine follow-up method are experienced by healthcare facilities, healthcare workers, and even patients and citizens in general. From a doctor’s point of view, this method means being able to see more patients, even outside their geographical area, and extending their hours of availability. Another significant benefit is the reduction of “no-show” rates, i.e., missed or postponed appointments. From the patients’ point of view, this method gives not only the patients but also their families an active and proactive role. From a healthcare perspective, it is a valuable tool for managing healthcare workflows, individual case management, prioritization, and decision making.

This is where technological innovation becomes imperative. The devices identified allow patients to acquire high-quality trichoscopic images directly from their homes. This not only reduces the need for frequent clinic visits, but also improves accessibility of care for patients living in remote areas or for those who have difficulty traveling to the clinic. The use of these devices is based on two fundamental pillars: ease of use and the diagnostic accuracy of the images collected. Thanks to intuitive design and user-friendly interfaces, patients can easily take trichoscopic photographs that capture crucial details for diagnosis. The quality of these images has been deemed by our team of expert dermatologists to be comparable to those obtained during in-clinic visits, allowing doctors to make accurate and timely assessments. A critical aspect of this approach is patient training. With proper instruction provided through procedure instructions, step-by-step guides, and continuous support, patients can become integral to their care process. The ability to collect reliable diagnostic images not only empowers patients, but also actively involves them in monitoring their condition. This sense of participation can enhance their adherence to treatment and overall satisfaction with the care received. Moreover, telemedicine, integrated with the use of these devices and a dedicated chatbot, has enabled more rapid and efficient communication between patients and doctors. In conclusion, the adoption of portable trichoscopic devices represents a significant breakthrough in the management of alopecia. It is our duty to continue exploring and developing these technologies, ensuring that every patient can access high-quality care, regardless of geographical or logistical barriers. It must be said, however, that this technique is not without its drawbacks, one of which is that the degree of diagnostic confidence experienced by the dermatologist is linked to the quality of the image transmitted by the patient. An additional risk of this methodology is the loss of a holistic view of the patient in favor of focusing on a single lesion. However, these drawbacks are partially overcome by the cooperation of the patient, who is made aware of the macroscopic and microscopic characteristics of the pathology from which he/she suffers and is, thus, able to identify lesions appearing in other areas. Another limitation is that this method can only be applied to subjects with basic cultural and technological skills. Our study focuses on a young population (subjects under 60 years of age), but we do not exclude the possibility of extending the age range with the help of a family member accustomed to using technological devices. In addition, the follow-ups for patients with established diagnoses at the first outpatient visit were evaluated; therefore, it was not possible to assign a diagnostic role to the patient, but only one of control and home management of therapy, as well as modifying the timing of control visits.

However, we are confident that this method can also be used for diagnostic purposes, given the ability to identify peculiar lesions, by performing the first visit remotely.

The ultimate goal is not to supplant traditional healthcare practices, primarily based on direct doctor–patient relationships, but to complement and enhance them, thereby improving service effectiveness and efficiency [12,13].

In addition, the results suggest that with appropriate guidance and support, provided by our instructions for patient procedures in the form of flowcharts (Figure 1), patients can effectively contribute to their diagnostic process.

## 5. Conclusions

In this contemporary digital era, technology is revolutionizing our engagement with healthcare and medicine. Telemedicine allows people to access quality medical care regardless of their geographical location or their chronic illnesses or disabilities.

We conducted a clinical study that showed good compliance and good clinical assessment in patients affected by alopecia areata who were followed via telemedicine.

Although our work is still at an embryonic stage and only a limited number of cases have been evaluated, we believe that the results obtained are encouraging and that there is ample room for global growth in the future through collaborative systems that can be applied on a large scale.

## Figures and Tables

**Table 1:** Systemic approach for the development of the chatbot capable of conducting monthly follow-ups with patients.

STEP	DESCRIPTION
Defining the bot’s objectives	Prior to commencing programming, it was imperative to delineate the bot’s objectives, including desired patient information, reminders, and responses.
Creating a Business account on Messenger Apps	Establishing a Chat bot necessitated the creation of a Business account on Messenger apps, facilitating the management of messages and access to enhanced features.
Selecting a development platform	Numerous development platforms offer tools for creating chat bots, enabling the formulation of conversation flows, response management, and data analysis through programming languages.
Constructing the conversation flow	the bot’s conversation flow was devised after selecting the development platform, outlining potential responses based on patient queries, such as directing patients to upload photos or laboratory results via accessible links.
Configuring reminders and implementing data collection	Reminders for crucial appointments, monthly follow-ups, and quality-of-life assessments were programmed into the bot; the bot collects patient information, monthly photographs, laboratory results, treatment efficacy, and adverse effects to facilitate healthcare professionals; monitoring of patient progress.
Testing and refining the bot	Rigorous testing with patients was conducted to assess the bot’s efficacy and usability, allowing for refinements to the conversation flow and overall performance.

**Table 2:** The chatbot manages the monthly follow-up of patients.

STEP	ESCRIPTION
Quality of life, visit and treatment response questionnaires	On the first day, the bot sends a link for patients to answer follow-up questions about quality of life, quality of visit and treatment response. Three questionnaires were used: SKINDEX - BDI-I HADS 4.
Analysis of answers	The bot analyzes patient responses and may pose further questions for additional investigation based on the answers provided.
Collect photos and lab tests	The bot furnishes a link through which patients input lab test results and are guided in capturing photos. Photos collected consist of four macro-photos taken with a mobile phone camera and four micro-photos taken with the micro camera.
Treatment update	Based on the information collected by the bot, the attending physician can view the material uploaded by the patient and decide to update the patient’s treatment.
Finish	The bot provides a farewell remark.
Loop	If the patient continues the follow-up, the bot will repeat all steps until the end of the follow-up.
End of follow-up	If the patient does not wish to continue the follow-up, the bot greets the patient and archives the information collected.

## Data Availability

The raw data supporting the conclusions of this article will be made available by the authors on request.
